# Limited Link between Oxidative Stress and Ochratoxin A—Induced Renal Injury in an Acute Toxicity Rat Model

**DOI:** 10.3390/toxins8120373

**Published:** 2016-12-14

**Authors:** Liye Zhu, Tao Yu, Xiaozhe Qi, Jing Gao, Kunlun Huang, Xiaoyun He, Haoshu Luo, Wentao Xu

**Affiliations:** 1 Beijing Advanced Innovation Center for Food Nutrition and Human Health, College of Food Science & Nutritional Engineering, China Agricultural University, No. 17 Tsinghua Donglu, Beijing 100083, China; zlyhome@163.com (L.Z.); anhuiyutao@gmail.com (T.Y.); qixiaozhe0706030224@126.com (X.Q.); gaojingsienna@gmail.com (J.G.); huangkl009@sina.com (K.H.); raininghe@163.com (X.H.); 2 State key Laboratory of Agrobiotechnology, Department of Animal Physiology, College of Biological Sciences, China Agricultural University, Beijing 100193, China; 3 The Supervision, Inspection and Testing Center of Genetically Modified Organisms, Ministry of Agriculture, Beijing 100083, China; 4 Beijing Laboratory of Food Quality and Safety, Beijing 100083, China

**Keywords:** Ochratoxin A, acute toxicity, nephrotoxicity, oxidative stress, DNA damage

## Abstract

Ochratoxin A (OTA) displays nephrotoxicity and hepatotoxicity. However, in the acute toxicity rat model, there is no evidence on the relationship between OTA and nephrotoxicity and hepatotoxicity. Based on this, the integrated analysis of physiological status, damage biomarkers, oxidative stress, and DNA damage were performed. After OTA treatment, the body weight decreased and AST, ALP, TP, and BUN levels in serum increased. Hydropic degeneration, swelling, vacuolization, and partial drop occurred in proximal tubule epithelial cells. PCNA and Kim-1 were dose-dependently increased in the kidney, but Cox-2 expression and proliferation were not found in the liver. In OTA-treated kidneys, the mRNA expressions of *Kim-1*, *Cox-2*, *Lcn2*, and *Clu* were dose-dependently increased. The mRNA expressions of *Vim* and *Cox-2* were decreased in OTA-treated livers. Some oxidative stress indicators were altered in the kidneys (ROS and SOD) and livers (SOD and GSH). DNA damage and oxidative DNA damage were not found. In conclusion, there is a limited link between oxidative stress and OTA-induced renal injury in an acute toxicity rat model.

## 1. Introduction

Ochratoxin A (OTA) is a mycotoxin that is produced by several fungal species in the *Aspergillus* and *Penicillium* genera [1,2,3]. OTA displays nephrotoxicity [4], hepatotoxicity [1], carcinogenicity [5], and neurotoxicity [6] in mammals. There are some studies that have revealed the mechanisms of OTA, such as, protein synthesis inhibition [7], lipid peroxidation [8], mitochondrial function inhibition [9], DNA methylation [10], miRNA regulation [11], the changes of enteric microorganism [12], renal cortex fibrosis, and epithelial-to-mesenchymal transition [13,14] etc. Some researchers also suggested that the toxicity is related to oxidative stress, and other researchers insist that DNA damage, including DNA adduct formation and DNA strand breaks, are responsible [15,16,17]. Which one of the mode of actions underlying OTA-induced acute nephrotoxicity is the key role? It still needs to be explored.

Previous studies demonstrated that OTA-induced cytotoxicity in vitro is highly correlated with the induction of oxidative DNA damage [1,18,19,20,21,22], and OTA treatment reduces cellular antioxidant defenses [21,23,24]. This proposal is strengthened by studies showing that antioxidants counteract OTA-mediated cytotoxicity [25,26].

However, until now, the results in vivo have been controversial. Several animal models were used to study OTA toxicities, but these studies were primarily based on the time and dose of a 2-year study of the National Toxicology Program (NTP) [27]. Most of these studies focused on the changes in medication treatment rather than the complete toxicity of OTA. The previous studies demonstrated oxidative damage in rats treated with 250 μg/kg OTA for 4 weeks [28] and 120 μg/kg OTA for 8 weeks [29]. However, DNA damage was not explored. Recently, DNA double-strand breaks and large deletion mutations were found in rats treated with OTA at 70, 210 or 630 µg/kg/day via gavage for 4 weeks [16]. However, oxidative damage was not explored in detail. Previous acute toxicity studies primarily focused on physiological and pathological changes. We previously explored oxidative damage and DNA damage in rats treated with carcinogenic doses (0, 70 or 210 μg/kg b.w.) of OTA for 4 or 13 weeks [30], and showed a trend of rat renal carcinogenicity with limited induction of oxidative stress responses.

Based on these studies, in the acute toxicity rat model, male wistar rats were fed with OTA for 7 days. Serum biochemical parameters, physiology, pathology, oxidative damage, and DNA damage were all detected. Based on these parameters, the relationship of oxidative damage and OTA-induced hepatotoxicity and nephrotoxicity were explored.

## 2. Results

### 2.1. OTA Affects the Physiological Status of Rats

**Figure 1:**
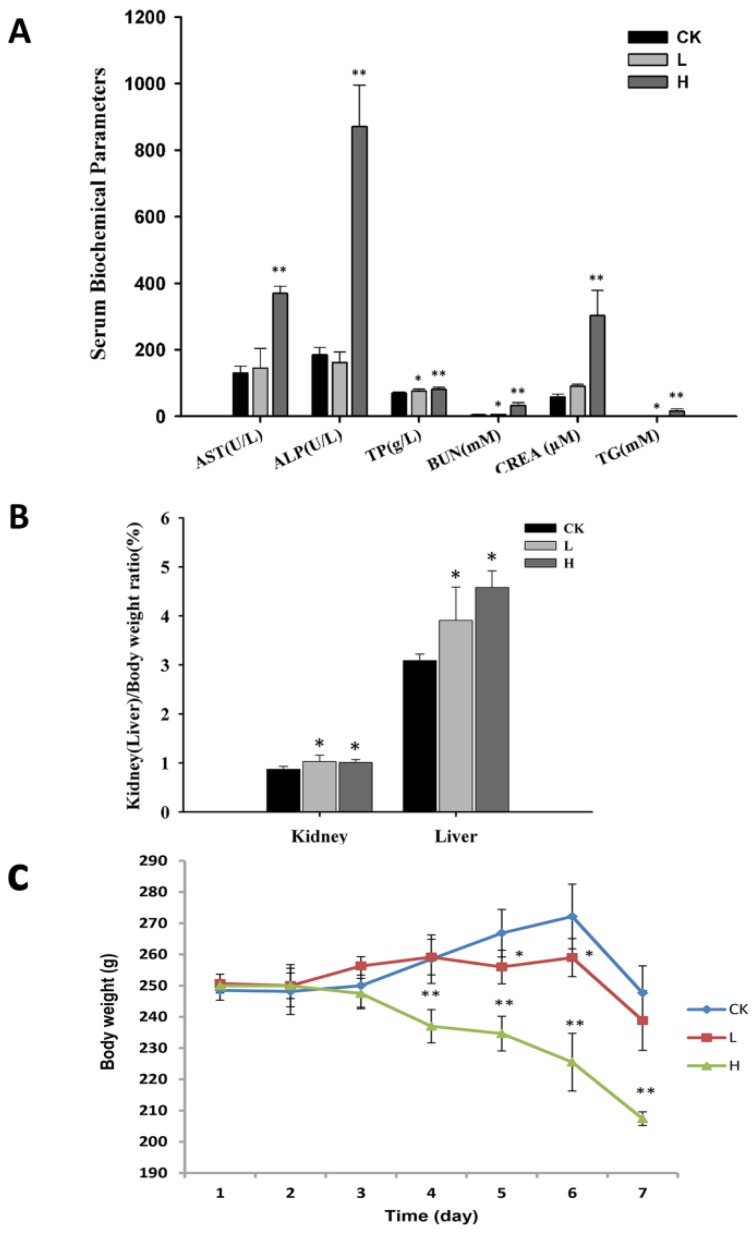
(**A**) The serum biochemical parameters of rats in different OTA administration groups; (**B**) The ratios of kidney (liver) and body weight were detected; (**C**) The body weights were detected in three groups. Male Wistar rats were treated with OTA (0, 1 or 4 mg/kg b.w.), denoted as CK, L, and H group, respectively for 7 days. The data are presented as the mean ± SD (*n* = 6). * *p* < 0.05, ** *p* < 0.01.

All rats lost weight after 7 days of OTA administration. The H group (4 mg/kg) exhibited decreased weight compared to the L group (1 mg/kg) and control group (corn oil) from the fourth day of the experiment (*p* < 0.05). The L group only showed a decrease compared to the control group on days 5 and 6 (*p* < 0.05) (Figure 1C).

The ratios of kidney and liver to body weight in H group compared to the control group exhibited significant increases. Also the ratios of brain, renicapsule, and testis to body weight increased significantly while the thymus showed a significant decrease. In the L group, only the ratios of brain, liver, and kidney to body weight compared to the control group increase (Figure 1B and Table 1). Notably, only the ratio of brain to body weight was dose-dependent. Moreover, the organ coefficient of the brain also increased with OTA dose in our experiment.

AST, ALP, GLU, BUN, CREA, TP, TG, and LDH in the H group showed a significant increase compared to the L and control groups (*p* < 0.05). However, the L group only showed increases in TP, ALB, and BUN compared to the control group (*p* < 0.05) (Figure 1A and Table 2). CREA in serum and BUN are the indicators of kidney health. CREA can be measured by product of muscle metabolism that is excreted unchanged by the kidney. BUN is formed in the process in whichthe liver produces urea. The increased levels of CREA and BUN in the L and H groups revealed an impairment of renal function. ALP, AST, and ALB are biomarkers of hepatic injury [31]. ALP and ALB increases in these proteins were only found in the H group. ALB increased in both L and H group. These results indicate that hepatic injury occurred in the H group.

### 2.2. OTA Caused Kidney Damage in Rats

**Figure 2:**
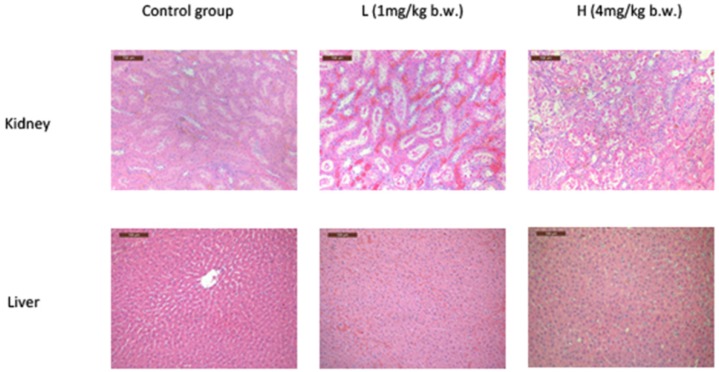
H & E stained kidney and liver sections after OTA treatment. Rats were treated with OTA (0, 1 or 4 mg/kg b.w.), denoted as CK, L, and H group, respectively for 7 days. Original magnification = 200×.

Denaturalization and necrosis in the epithelial cells of renal tubules were found in both L and H groups (Figure 2A). The denaturalization and necrosis were primarily distributed in the layer of cortex and outer medulla in the L group, but this damage expanded to the inner medulla in the H group. The glomerulis were swollen in the cortex layer in the L and H groups. Moreover, in the L group, renal tubular dilated in the layer of cortex and outer medulla shallow. Also in the cortex, hydropic degeneration and partial drop occurred in epithelial cells of the proximal tubule. There are a small amount of protein and cellular casts in the outer medulla shallow. In the H group, hydropic degeneration, swelling, vacuolization, and partial drop occurred in the proximal tubule epithelial cells of the cortex and medulla. A little karyopyknosis, karyorrhexis, and karyolysis occurred locally in the proximal renal tubular epithelial cell (Figure 2A). The pathological results showed an expansion of OTA-induced renal damaged. Notably, even the animals that received OTA at a dose of 4 mg/kg b.w. did not show any significant pathological differences in the liver compared with the control group (Figure 2B).

**Figure 3:**
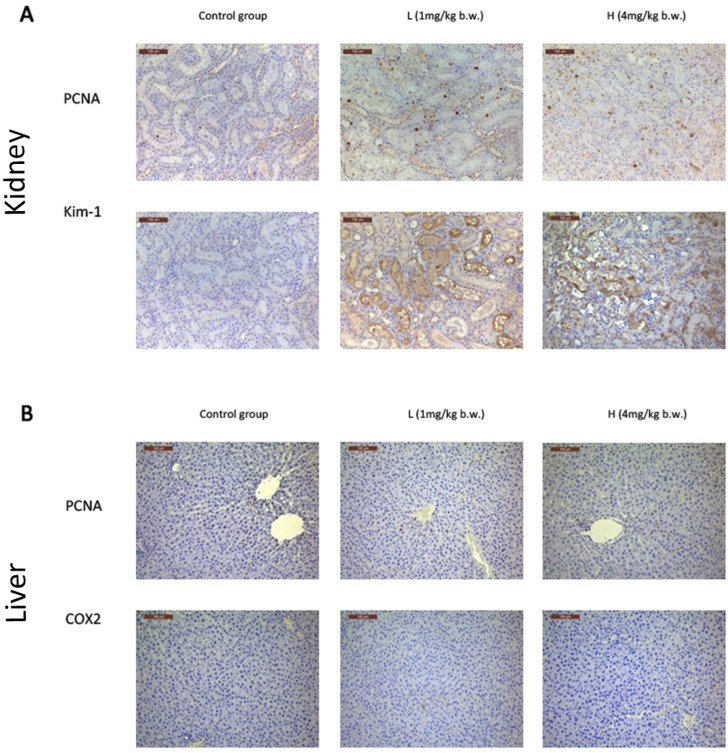
Immunohistochemical staining. (**A**) The immunohistochemical staining of PCNA and Kim-1 in the kidney; (**B**) The immunohistochemical staining of PCNA and Cox-2 in the liver. Rats were treated with OTA (0, 1 or 4 mg/kg b.w.), denoted as CK, L, and H group, respectively for 7 days. Original magnification = 200×.

PCNA is a 36-kD nuclear protein that is associated with the cell cycle [32]. L and H group rats showed dose-dependent increases in positive PCNA signals in the kidney, which indicates that an increase of cell proliferation occurred in the kidney (Figure 3A). However, no significant changes were observed in the liver (Figure 3B).

**Figure 4:**
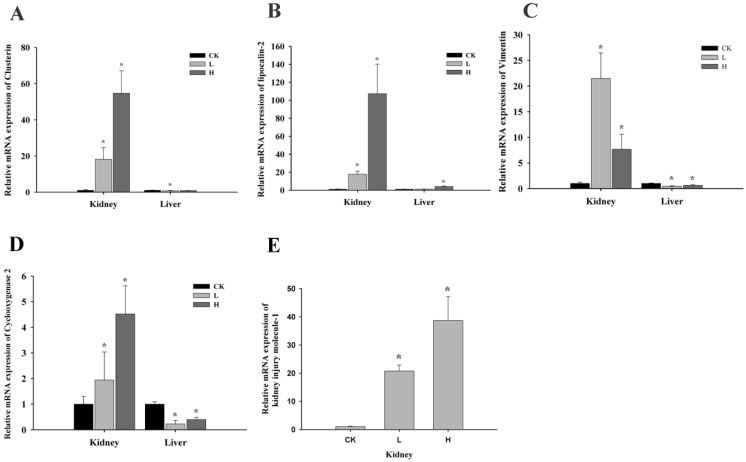
Changes in the mRNA expression of *Clu*, *Lcn2*, *Vim,* and *Cox-2* in kidneys and livers of male Wistar rats dosed with OTA for 7 days. *Kim-1* was only detected in kidneys; C: control group (0 mg/kg b.w.); L: low-dose group (1 mg/kg b.w.); H: high-dose group (4 mg/kg b.w.). **A**: *Clu*; **B**: *Lcn2*; **C**: *Vim*; **D**: *Cox-2*; **E**: *Kim-1*. Data are presented as the means ± SD (*n* = 6). * *p* < 0.05

Kim-1 is a specific and sensitive biomarker of kidney injury [33]. The immunochemistry of Kim-1 was also detected in the kidneys of L and H groups. Positive signals were primarily located in tubular epithelial cell membranes, with sparse expression in the cytoplasm (Figure 3A). The mRNA expression of *Kim-1* demonstrated a dose-dependent increase in the kidney (Figure 4E).

### 2.3. OTA Induced Putative Biomarkers of Nephrotoxicity

In the kidney, the mRNA expression of *Clusterin* (*Clu*), *lipocalin-2* (*Lcn2*), Cyclooxygenase2 (*Cox-2*), and kidney injury molecule-1 (*Kim-1*) showed dose-dependent increases in all groups (*p* < 0.05) (Figure 4A,B,D,E). However, the mRNA expression of *Vimentin* (*Vim*) increased 21-fold in the L group but only 7-fold in the H group compared to control group (*p* < 0.05) (Figure 4C).

In the liver, the mRNA expressions were also measured as a comparison with the kidney. *Lcn2* showed a 4-fold increase in the H group compared to control group (*p* < 0.05) (Figure 4B). Notably, *Vim* and *Cox-2* expression decreased in the L group. However compared with the L group, they increased again in the H group (*p* < 0.05) (Figure 4C,D).

### 2.4. Oxidative Stress Plays a Limited Role in OTA-Induced Rat Acute Kidney Injury

Reactive Oxygen Species (ROS) have several forms: superoxide radical (O^2−^), hydrogen peroxide, hydroxyl radical, hydroxyl ion, and nitric oxide. Cells have a variety of defense mechanisms to ameliorate the harmful effects of ROS [34].

**Figure 5:**
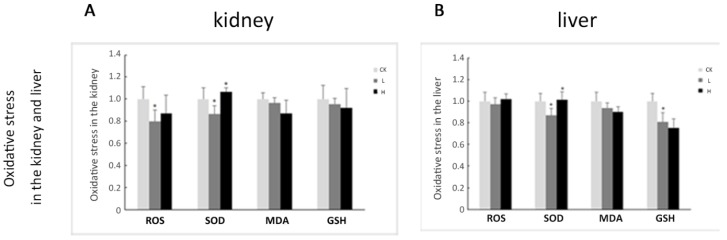
Expression of ROS, SOD, MDA, and GSH in kidneys and livers of male Wistar rats treated with OTA for 7 days. **A**: The detection of oxidative stress in the kidney; **B**: The detection of oxidative stress in the liver. CK: control group (0 mg/kg b.w.); L: low-dose group (1 mg/kg b.w.); H: high-dose group (4 mg/kg b.w.). Data are presented as the means ± SD (*n* = 6). * *p* < 0.05.

SOD activity in the kidney and liver decreased approximately 13% in the L group compared to the control group (*p* < 0.05), and increased again in the H group compared to the L group (*p* < 0.05). However, SOD activity in the H group was not significantly different from the control (Figure 5). MDA activity was not significantly different in the kidney and liver between groups. Total ROS decreased approximately 20% in the kidney of the L group compared to the control group (*p* < 0.05). GSH content in the liver decreased approximately 19% in the L group compared to the control group (*p* < 0.05) (Figure 5). These markers reveal that OTA slightly affects rat acute kidney injury due to oxidative stress.

### 2.5. DNA Damage Was Not Observed in Acute Toxicity

**Figure 6:**
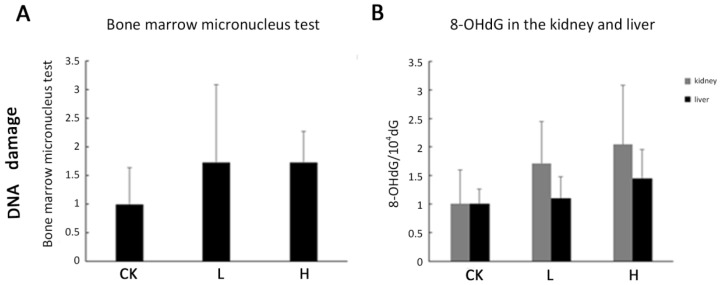
DNA damage and DNA oxidative damage. Male Wistar rats were treated with OTA (0, 1 or 4 mg/kg b.w.), denoted as CK, L, and H group, respectively for 7 days. (**A**) DNA damage was detected by bone marrow micronucleus test; (**B**) 8-OHdG levels were determined in kidneys and livers. Data are presented as the means ± SD (*n* = 6).

DNA damage was not observed using the comet assay under our experimental conditions in OTA-treated livers and kidneys compared to the controls (data not shown). The marrow micronucleus test (MNT) also did not reveal significant difference between OTA-treated and control rats (Figure 6A). 8-OHdG is formed when DNA is damaged by hydroxyl radicals, and it is always used to detect DNA damage. 8-OHdG was measured after the enzymatic hydrolysis of DNA. However, there was no significant difference between OTA-treated livers and kidneys compared to controls (Figure 6B). We speculated that DNA damage is not produced and there is limited link between acute renal injury and oxidative stress.

## 3. Discussion

Based on the previous studies, the mechanisms of OTA were explored [7,8,9,35,36,37]. Oxidative stress has an important role in vitro. While in in vivo studies, it is controversial. However, until now, the acute toxicity rat model has been less studied on OTA-induced toxicity. Whether oxidative stress is one of the reasons of renal injury in an acute toxicity rat model, is still indefinite. Based on this, integrated analyses of physiological status, kidney and liver damage, biomarkers, and oxidative damage, and DNA damage were performed in this study to clarify the acute toxicity of OTA.

In the study, pathological and immunohistochemical results demonstrated that OTA induced kidney damage, but not liver damage. These results were consistent with some studies. For example, before the major pathological changes, the increasing mRNA expressions of Kim-1, Lcn2, Clu, and Vim revealed injury in various models of acute kidney injury. Among these, Kim-1 is the specific and sensitive biomarker of kidney injury. Lcn2 is regarded as the biomarker of acute kidney injury. Clu is also induced in a variety of acute and chronic experimental renal diseases [38]. Furthermore, Kim-1 and Lcn2 were specifically induced in animal models and human renal diseases that involve acute injury of the proximal tubule epithelium [39,40]. Cox-2 is a dramatically up-regulated gene during injury, inflammation, and proliferation [41]. Increased Kim-1 and Lcn2 expression were also observed in the kidneys of 4 weeks and 13 weeks OTA-treated animals [42]. In addition, positive PCNA signals increases in the kidney after OTA treatment. This result is consistent with previous researches. Mally et al. [43] also found the increased expression of PCNA in the kidney but not the liver of rats. Also, Rached et al. [44] stated that the early effects of OTA treatment consisted of cell loss accompanied by increased cell proliferation and prominent nuclear enlargement within the straight segment of the proximal tubule epithelium (P3) in the OSOM. Besides, proliferation also has been regarded as one of the indicators of OTA-induced tumor in Qi et al. [30]. Based on this, the proliferation in the kidney may be the mode of action of early effects of OTA-induced toxicity. Also, blood biochemistry and putative biomarkers revealed kidney damage (CREA and BUN) and liver damage (ALP, AST, and ALB). Among liver damage biomarkers, only ALB increased in L and H groups. ALB, produced by the liver, occurs dissolved in blood plasma and is the most abundant blood protein in mammals. Albumin functions primarily as a carrier protein for steroids, fatty acids, and thyroid hormones in the blood and plays a major role in stabilizing extracellular fluid volume by contributing to the oncotic pressure of plasma. However, there is no significant difference of albumin levels between L and H groups. So this change should be the stress response of the rats. On the other hand, some studies have indicated that OTA is bound to serum albumin to exert its toxicity role [45,46]. This also can explain the elevation of the level of albumin in serum. Pathological change always occurs with severe damage, and OTA treatment for longer than 4 weeks induced kidney and liver damage [30,44].These results indicated that blood parameters and biomarkers suggest damage earlier than pathological observations. This result is also consistent with our long-term experiments [30]. 

Many in vitro studies reported that oxidative stress was the proposed cause that enables OTA treatment to generate ROS, which results in oxidative DNA damage [1,18,19,20]. There are three types of evidence. First, DNA double-strands breaks were detected in OTA-treated HK-2 cell by comet assay. Second, oxidative DNA damage was also observed. Third, ROS was increased in OTA-treated HK-2 cell.

However, the oxidative stress response is controversial in in vivo studies [30,42,44]. ROS is produced as a normal product of cellular metabolism. However, t excessive amounts can cause deleterious effects. ROS in our experiment was only decreased in the kidney in the L group compared to the control group, without change in the liver. These results indicated that oxidative stress occurred slightly in OTA-induced renal injury. GSH is an important antioxidant. It can prevent the damage induced-by ROS. It has two types, including G-SH and G-S-S-G while in our study, there are no significant difference in kidney and liver after OTA-treated. SOD plays an antioxidant role in the organism. SOD is necessary because superoxide reacts with sensitive and critical cellular targets, such as NO radical. In our study, the changes of SOD are consistent with the changes of ROS in kidney and liver. High-dose OTA stimulation might better induce SOD activity in the kidney and liver as a defense mechanism to improve the harmful effects of ROS.

OTA toxicity varies across organs, doses, and time. The results of the studies showed that OTA-induced oxidative damage was different in liver and kidney. These differences are likely due to the different defense mechanisms, GSH activity in the liver, and SOD activity in the liver and kidney. Oxidative damage was not dose-dependent in short-term treatment. Bolt et al. reported that chemical-induced DNA damage through indirect mechanisms such as ROS production may exist as a practical threshold [47]. The different dose effects of OTA require further exploration to clarify whether there is a threshold value to oxidative stress and the involved defense mechanisms.

MDA is produced by ROS degrading polyunsaturated lipids to form MDA. It is used as a biomarker to measure the level of oxidative stress in an organism. MDA levels were not different after OTA (0–2 mg/kg) treatment for 4–48 h [48], but MDA increased after 250 µg/kg of OTA treatment for 4 weeks [28] and 120 µg/kg OTA treatment for 8 weeks [29]. These results suggest that MDA formation is not the prime oxidative stress response of OTA-induced acute toxicity. However, no difference in MDA level in the kidney and liver in our 7-day experiment was found. It proves that oxidative stress was not the toxic mechanism.

Articles have reported that OTA can induce the break of DNA strand in the kidney [49,50,51,52]. However, one study also showed that 8-OHdG levels in the kidney after 13 weeks of OTA exposure were not significantly different compared with the control [53]. Rats treated with 1 mg/kg OTA for 24 h did not exhibit the increase of 8-OHdG levels in rat kidney [48]. A recent study also showed that OTA has the potential to accumulate DSBs because OTA caused a 5-fold increase in mutation frequencies in F344 rats treated with OTA for 4 weeks compared to the control animals [54]. The same evidence was found in the comet assay and the expression of γ-H2AX, which is a promising marker of DSBs [55,56]. OTA-induced karyomegaly and subsequent carcinogenicity targeting the OSOM may be unrelated to alterations of the redox state [57]. These results suggest that the genotoxic potential of OTA may not be directly related to oxidative stress. 8-OHdG is an indicator of oxidative DNA damage. The relatively independent mechanism of OTA-induced oxidative damage and DNA damage might explain the undetected differences in 8-OHdG levels between OTA-treated animals and control animals in a subchronic toxicity test and our acute toxicity test. The oxidative DNA damage may be caused after an accumulation of oxidative damage over a longer period of time.

In conclusion, our results demonstrated that apparent kidney damage occurs from different perspectives. Different mechanisms of oxidative stress are involved in the kidney and liver. OTA doses and treatment duration may exert different oxidative damage. Oxidative damage and DNA damage caused by OTA may not be directly related or synchronized because of the different pattern of changes. Our experiment provides a new perspective about the relationship between oxidative stress and acute renal injury in an acute toxicity rat model while the results revealed that oxidative stress plays a limited role.

## 4. Materials and Methods

### 4.1. Ethics Statement

All procedures in experiments were approved by the Animal Care Ethics Committee of the China Agricultural University (permission number: 120020, 9 October 2014). Animal studies were conducted in the Supervision & Testing Center for Genetically Modified Organisms Food Safety, Ministry of Agriculture (Beijing, China) (license number SYXK (Beijing, China) 2010–0036).

### 4.2. Animals

Male Wistar rats (7 weeks old, male) were fed at the Supervision and Testing Center for GMOs food safety, Ministry of Agriculture (Beijing, China). The animal room was maintained at a temperature of 22 ± 2 °C, relative humidity of 40%–70%, artificial illumination (fluorescent light) on a 12 h light/dark cycle, and air exchange of 15 times/h. Rats were gavaged with OTA dissolved in corn oil (Aladin, Shanghai, China) at the respective doses (0, 1 or 4 mg/kg b.w.), denoted as CK, L, H, groups, respectively for 7 days. Body weights were recorded daily. Rats were anesthetized using chloral hydrate (6%, 5 mL/kg, i.p.), and blood was obtained from the orbital sinus. Serum biochemical parameters were measured using a Hitachi 7020 automatic biochemical analyzer (Hitachi, Tokyo, Japan). Alanine aminotransferase (ALT), aspartate aminotransferase(AST), total serum protein (TP), alkaline phosphatase (ALP), albumin (ALB) creatinine (CREA), cholesterol (CHOz), triacylglycerol (TG), glucose (GLU), blood urea nitrogen (BUN), and lactate dehydrogenase (LDH) in serum were measured. The brain, liver, spleen, heart, thymus, kidney, renicapsule, and testis were weighed. The samples were partially stored at −80 °C for further analysis and partially prepared for routine histopathology and the immunohistochemistry.

### 4.3. Immunohistochemistry

The paraffin sections (5 μm) of kidney and liver were prepared. Sections were deparaffinized, rehydrated, and washed in PBS. Endogenous peroxidase activity was blocked with 3% hydrogen peroxide for 30 min. Also antigen retrieval was performed using 0.01 M citrate buffer (pH 6.0) in a microwave oven. Immunostaining was performed following the streptavidin biotin-peroxidase complex method. Briefly, 10% goat serum for proliferating cell nuclear antigen (PCNA) and donkey serum for kidney injury molecule-1 (Kim-1), Cyclooxygenase-2 (COX-2) (Santa Cruz, CA, USA) were used as the blocking solution. Primary antibodies, rat PCNA, rat Kim-1, and rat COX-2 (Santa Cruz, CA, USA), were incubated overnight at 4 °C and washed with PBS. Then secondary antibodies, Goat Anti-Rat IgG H&L (GARB) and Donkey Anti-Goat IgG H&L (DAGB) (Santa Cruz, CA, USA), were incubated for 2 h at room temperature and washed with PBS. Sections were incubated with streptavidin-horseradish peroxidase (HRP) for 2 h. Finally, the sections were stained using DAB (3,3’-diaminobenzidine) and hematoxylin. After mounting, digital photographs were obtained using a Leica DM2500.

### 4.4. RNA Extraction and Quantitative Real-Time Polymerase Chain Reaction (qRT-PCR)

Total RNA was isolated from the liver and kidney using the modified TRIzol method. The cDNA was synthesized using the Reverse Transcription System (Promega, Madison, WI, USA) according to the manufacturer's protocol using oligo (dT) as primers. The real-time PCR reaction was performed using the RealMasterMix (SYBR green I) (Tiangen, China) in a Bio-Rad CFX96 real-time PCR System (Bio-Rad, Irvine, CA, USA). The thermal cycling program was set as follows: 95 °C for 5 min, followed by 40 cycles of 95 °C for 10 s, and 60 °C for 1 min. A melting curve analysis was conducted following amplification by heating from 65 °C to 95 °C in increments of 0.5 °C for 5 s to determine the specificity of the PCR reactions. The fluorescence signal was collected at the end of the elongation step for each cycle. The control gene chose *β-actin*. The primer sequences are shown in Table 3. Relative gene expressions were evaluated using the 2^−ΔΔCT^ method [58].

### 4.5. Determination of Oxidation-Related Parameters

Approximately 0.1 g of tissue homogenates from livers and kidneys was transferred into a pre-weighed tube containing 500 μL of phosphate-buffered saline (pH7.4) for ROS, malonaldehyde (MDA), reduced glutathione(GSH), and superoxide dismutase (SOD) analyses. The mixture was mixed well and centrifuged at 2500 r/min for 20 min. Aliquots of the supernatant were used immediately. SOD activity and the content of ROS, MDA, GSH were measured using rat ELISA Kits (Beijing FangchengShengwuKeji Inc., Beijing, China).

### 4.6. Analysis of 8-Hydroxydeoxyguanosine (8-OHdG)

DNA extraction and digestion was performed according to a previous report [59]. Samples were homogenized using lysis buffer. The mixture was centrifuged at 10,000× *g* for 20 s at 4 °C. The deposit was dissolved in 200 μL of enzyme reaction buffer and treated with RNase and protease K. DNA was obtained by washing in 2-propanol and ethanol followed by centrifugation. DNA was dissolved in 20 μM sodium acetate buffer, pH 4.8, and incubated with 4 μL of nuclease P1 (2000 U/mL) at 70 °C for 15 min. Then, 20 μL of 1.0 M Tris-HCl buffer, pH 8.2, was added, and the sample was incubated with 4 μL of alkaline phosphatase (2500 U/mL) at 37 °C for 60 min. The digested DNA samples were acquired after the addition of 20 μL of 3.0 M sodium acetate buffer, pH 5.1 [53]. The mixture was filtered through a 0.22 μm Ultra free-MC filter (Millipore, Billerica, MA, USA) to remove proteins. Analysis of the DNA base composition was performed using an HPLC/ESI/MS/MS system (Agilent, Santa Clara, CA, USA) and an Agilent Poroshell 120 EC-C18 3.0 (50 mm × 2.7 μm). Mass spectrometric detection was performed using an Agilent 6460 triple quadrupole tandem mass spectrometer (Agilent, Palo Alto, CA, USA) coupled with an electrospray ionization source. The formation of 8-OHdG was calculated as 8-OHdG/10^5^ dG [22].

### 4.7. Comet Assay

The alkaline (pH >13) single-cell microgel electrophoresis technique assay (comet assay) was used to detect DNA damage. Frozen liver and kidney tissues were thawed at room temperature during preparation, placed in cylindrical sieves and immersed in 1.5 mL ice-cold Merchant’s medium [60]. Tissues were homogenized by moving a plunger up and down several times to press the tissue through the sieves. This process produced a cell suspension. The cells were trypsinized and suspended in PBS at a density of approximately 1 × 10^5^ cells/mL. The slides were coated with 100 μL of 1% normal melting agarose (Amresco, Solon, OH, USA). The cell pellets were resuspended with 75 μL of 0.6% low-melting agarose (Amresco, Solon, OH, USA) and applied to the prepared slides. The agarose was allowed to gel for 10 min before the cover slip was removed. The cells were lysed under alkaline conditions. The samples were lysed overnight at 4 °C in the dark.

The slides were placed in a horizontal gel electrophoresis chamber (Renner, Dannstadt, Germany), covered with alkali electrophores is buffer (pH >13.0), allowed to unwind for 20 min, and subjected to electrophoresis. The slides were washed with 0.4 M Tris buffer (pH 7.5), stained with 5 μg/mL propidium iodide (PI) (Beyotime, Shanghai, China) for 20 min and dried. Images were captured using an Olympus BX-51 fluorescent microscope (Olympus, Tokyo, Japan). At least 100 cells were randomly selected from each group and analyzed using the Comet Assay Software Project (CASP) 1.2.2 (CASPlab, Wroclaw, Poland) [22].

### 4.8. Statistical Analysis

The data were analyzed using an analysis of variance (ANOVA) or Student’s *t*-test, and the results were expressed as the means ± standard deviation (SD). Six rats were used per group. Each experiment was performed in triplicate. *p* < 0.05 was considered statistically significant.

## Figures and Tables

**Table 1:** The absolute weight of the organs after 0, 1 or 4 mg/kg b.w. of OTA treatment for 7 days. The H group showed significant increases compared to the control in the ratios of brain, renicapsule, and testis to body weight, and the thymus showed significant decreases. The L group only exhibited increases in the ratios of brain, liver, and kidney to body weight compared to the control group.

Group	Brain	Kidney	Liver	Spleen	Heart	Thymus	Adrenal Gland	Testis
CK	0.76 ± 0.05	0.87 ± 0.06	3.09 ± 0.13	0.26 ± 0.04	0.4 ± 0.03	0.22 ± 0.07	0.034 ± 0.005	1.3 ± 0.08
L	0.85 ± 0.04 ^a^	1.03 ± 0.13 ^a^	3.91 ± 0.68 ^a^	0.25 ± 0.04	0.44 ± 0.11	0.22 ± 0.05	0.041 ± 0.007	1.31 ± 0.12
H	0.94 ± 0.05 ^ab^	1.01 ± 0.06 ^a^	4.58 ± 0.34 ^a^	0.29 ± 0.08	0.42 ± 0.03	0.12 ± 0.04 ^b^	0.063 ± 0.004 ^ab^	1.58 ± 0.18 ^ab^

^a^ Significantly different from the control group at *p* < 0.05; ^b^ Significantly different from the L group at *p* < 0.05.

**Table 2:** The serum biochemical parameters, AST, ALP, TP, GLU, BUN, CREA, TG, CHOz, and LDH in the H group were significantly increased compared to the L group and control group (*p* < 0.05). The L group only showed increases in TP, ALB, and BUN compared to the control group (*p* < 0.05).

Content	Control	L	H
ALT (U/L)	30.67 ± 8.76	45.75 ± 32.4	63.33 ± 35.81
AST (U/L)	131 ± 20	145 ± 59	370 ± 21 ^ab^
TP (g/L)	70.35 ± 3.76	76.85 ± 4.94 ^a^	80.95 ± 6.43 ^ab^
ALB (g/L)	29.78 ± 1.22	33.77 ± 1.69 ^a^	33.53 ± 5.66 ^a^
ALP (U/L)	185 ± 22	162 ± 32	871 ± 125 ^ab^
GLU (mmol/L)	2.14 ± 0.73	2.24 ± 0.25	8.39 ± 0.94 ^ab^
BUN (mmol/L)	5 ± 0.9	6.3 ± 0.8 ^a^	33.3 ± 8.2 ^ab^
CREA (μmol/L)	58 ± 9	91 ± 6 ^a^	303 ± 76 ^ab^
CHOz(mg/dl)	1.29 ± 0.18	1.03 ± 0.28	3.07 ± 2.21
TG (mmol/L)	0.68 ± 0.24	0.76 ± 0.3	15.32 ± 7.25 ^ab^
LDH (U/L)	1182 ± 392	702 ± 454	2785 ± 803 ^ab^

^a^ Significantly different from the control group at *p* < 0.05; ^b^ Significantly different from the L group at *p* < 0.05.

**Table 3:** Primers of genes for qRT-PCR.

Gene Name	Forward Primer	Reverse Primer
*Clusterin*	CACTACGGGCCTCTGAGCTT	ACGTCCATGGCCTGTTGAG
*Vimentin*	GATGCTCCAGAGGGAGGAAG	AAGGTCAAGACGTGCCAGAG
*lipocalin2*	TCTGGGCCTCAAGGATAACAAC	AGACAGGTGGGACCTGAACCA
*Kim-1*	TGGCACTGTGACATCCTCAGA	GCAACGGACATGCCAACATA
*COX-2*	TGTATGCTACCATCTGGCTTCGG	GTTTGGAACAGTCGCTCGTCATC
*β-actin*	TCGTGCGTGACATTAAGGAG	AGGAAGGAAGGCTGGAAGAG

## Abbreviations

OTA	Ochratoxin A
AST	aspartate aminotransferase
ALP	alkaline phosphatase
TP	total serum protein
TG	triacylglycerol
BUN	blood urea nitrogen
ALT	alanine aminotransferase
ALB	albumin
CREA	creatinine
GLU	glucose
PCNA	proliferating cell nuclear antigen
Kim-1	kidney injury molecule-1
Cox-2	Cyclooxygenase 2
Lcn2	lipocalin-2
Clu	Clusterin
Vim	Vimentin
ROS	Reactive Oxygen Species
MNT	marrow micronucleus test
GSH	reduced glutathione
SOD	superoxide dismutase
MDA	malonaldehyde
PI	propidium iodide
LDH	lactate dehydrogenase
8-OHdG	8-hydroxydeoxyguanosine
CHO/CHOz	cholesterol
